# CAR T cells: engineered immune cells to treat brain cancers and beyond

**DOI:** 10.1186/s12943-022-01712-8

**Published:** 2023-01-31

**Authors:** Zoufang Huang, Saikat Dewanjee, Pratik Chakraborty, Niraj Kumar Jha, Abhijit Dey, Moumita Gangopadhyay, Xuan-Yu Chen, Jian Wang, Saurabh Kumar Jha

**Affiliations:** 1 Department of Hematology, Ganzhou Key Laboratory of Hematology, The First Affiliated Hospital of Gannan Medical University, Ganzhou, China; 2 Advanced Pharmacognosy Research Laboratory, Department of Pharmaceutical Technology, Jadavpur University, Kolkata, 700032 India; 3 Department of Biotechnology, School of Engineering & Technology, Sharda University, Greater Noida, Uttar Pradesh 201310 India; 4 Department of Life Sciences, Presidency University, 86/1 College Street, Kolkata, West Bengal 700032 India; 5 Department of Biotechnology, School of Life Science and Biotechnology, Adamas University, Barasat, Kolkata, West Bengal 700126 India; 6 Institute for Biotechnology, St. John’s University, Queens, New York, 11439 USA; 7 Department of Radiotherapy, the Affiliated Jiangyin People’s Hospital of Nantong University, Jiangyin, 214400 China; 8 Department of Biotechnology Engineering and Food Technology, Chandigarh University, Mohali, 140413 India; 9 Department of Biotechnology, School of Applied & Life Sciences (SALS), Uttaranchal University, Dehradun, 248007 India

**Keywords:** Brain cancer, CAR T cells, Clinical trials, Hematological cancer, Immunotherapy, Solid tumors, Tumor antigen

## Abstract

Malignant brain tumors rank among the most challenging type of malignancies to manage. The current treatment protocol commonly entails surgery followed by radiotherapy and/or chemotherapy, however, the median patient survival rate is poor. Recent developments in immunotherapy for a variety of tumor types spark optimism that immunological strategies may help patients with brain cancer. Chimeric antigen receptor (CAR) T cells exploit the tumor-targeting specificity of antibodies or receptor ligands to direct the cytolytic capacity of T cells. Several molecules have been discovered as potential targets for immunotherapy-based targeting, including but not limited to EGFRvIII, IL13Rα2, and HER2. The outstanding clinical responses to CAR T cell-based treatments in patients with hematological malignancies have generated interest in using this approach to treat solid tumors. Research results to date support the astounding clinical response rates of CD19-targeted CAR T cells, early clinical experiences in brain tumors demonstrating safety and evidence for disease-modifying activity, and the promise for further advances to ultimately assist patients clinically. However, several variable factors seem to slow down the progress rate regarding treating brain cancers utilizing CAR T cells. The current study offers a thorough analysis of CAR T cells’ promise in treating brain cancer, including design and delivery considerations, current strides in clinical and preclinical research, issues encountered, and potential solutions.

## Introduction

Brain tumor, in general, is a mass/growth of abnormal brain cells. Malignant brain tumors can either originate primarily in the brain or as secondary or metastatic brain tumors that have metastasized from other types of cancer. Brain cancer patients usually survive only up to 5 years, that too with a significant compromise regarding their quality of life. Present therapeutic options are rarely curative and present with many undesired toxic effects. The success in T cell immunotherapy across a wide variety of tumor types has ignited the hope that the immune system can be strengthened to enhance outcomes for patients with brain tumors [1, 2]. Re-engineered T cells with a new selectivity towards specific tumor antigen(s) are emerging prospects for brain cancer management. One of the newest and most promising cancer treatments, chimeric antigen receptor (CAR) T cell therapy, boosts the body’s immune system to combat cancer. The advancement of CAR T treatment has been made possible by the convergence of gene sequencing, growing genetic knowledge, new methods of genome manipulation, and the development of novel gene transfer technologies. Prominent success for CAR T cell therapy was achieved earlier with lymphoma, leukemia, and other hematological cancers [3]. Relying upon the principle of specific antigen recognition, CAR T cells bear promise regarding the treatment of different solid tumors, including malignant tumors in the brain.

Access of the immune system to the brain is carefully regulated, and the immunosuppressive nature of CNS has developed to defend against immunologic attack. Approaches to boost endogenous T cell immune responses to treat brain tumors have been clinically proven to be effective despite significant difficulties. They are created by re-engineering T cells from the patient in the laboratory to produce proteins on their surface known as CARs. CARs recognize and bind to specific proteins on the surface of cancer cells. CAR-transplanted T cells gain the capacity to detect and lyse specific cancer cells. When used to treat tumors with minimal tumor mutational burden, such as the majority of brain tumors, CARs can target tumors without altering how the major histocompatibility complex (MHC) expresses its antigens [4, 5]. The fact that so many researchers are testing these strategies against brain cancer in both preclinical and clinical settings is a hint of their intriguing potential. The promise of CAR T cells in the treatment of brain cancer is emphasized in this review, along with considerations for their design and delivery, the recent therapeutic findings, issues encountered, and potential remedies.

## Brain tumors: clinical constraints towards treatment

Brain tumors exhibit a poor prognosis and an extremely low patient survival rate. Combining the clinical features, genetic orientations, and degree of lethality, the World Health Organization (WHO) categorized CNS tumors into four grades [6]. Grade 1 refers to slow-growing benign tumors that heal after resection; grade 2 tumors are slow-growing tumors that often recur and sometimes tend to become malignant. Quickly proliferative grades 3 and 4 are histologically and cytologically malignant tumors, respectively. Every year, approximately 3 per 100,000 people worldwide acquire some form of brain tumors [7]. The classical therapeutic scheme of brain cancer includes surgical resection followed by chemotherapy and/or radiation therapy. However, a growing body of evidence revealed that the chances of relapse are very high for malignant brain tumors [8, 9].

The presence of the blood-brain barrier (BBB) limits the therapeutic efficacy of certain chemotherapeutic drugs by restricting their penetration into the brain. Endothelial tight junctions in combination with efflux transporters, such as P-glycoprotein (P-gp), peptide transport system 6 (PTS6), and breast cancer-resistant protein (BCRP) restrict the entry of most of the drug molecules into the CNS [8, 10]. Enzymes, such as aminopeptidases and endopeptidases potentially render the cargo ineffective. Moreover, binding with non-transporter proteins can minimize available drug(s). Angiogenic vasculatures originating from tumors make up the blood-brain tumor barrier (BBTB) that influences the chemotherapeutic potential of a drug by regulating tight endothelial connections; Moreover, extracellular matrices in the tumor microenvironment are frequently regulated by tumor cells to encourage chemoresistance, and tumor cell proliferation and differentiation. Summarily, the poor prognosis of brain cancer is a combined result of limited access to drug delivery cargos, multi-drug resistance, and the critical ability of residual malignant cells to recur.

Current therapeutic tools used to treat brain cancer suffer from certain limitations. In case of surgery, accurate delineation of tumor boundary is difficult for proper resection, thus complete removal of malignant cells is rarely achieved. Resistance, and inability to precisely reach the CNS resulting in non-specific toxicities to other organs and tissues apprehend promises of chemotherapy. Radiation tends to exert effects largely on peripheral cells only. In addition, similar to surgery, radiotherapy also suffers from inaccurate delineation. Immunotherapy has come up with promising outputs to boost the immune system and outperform immune suppression imposed by tumor cells [11, 12].

**Fig. 1:**
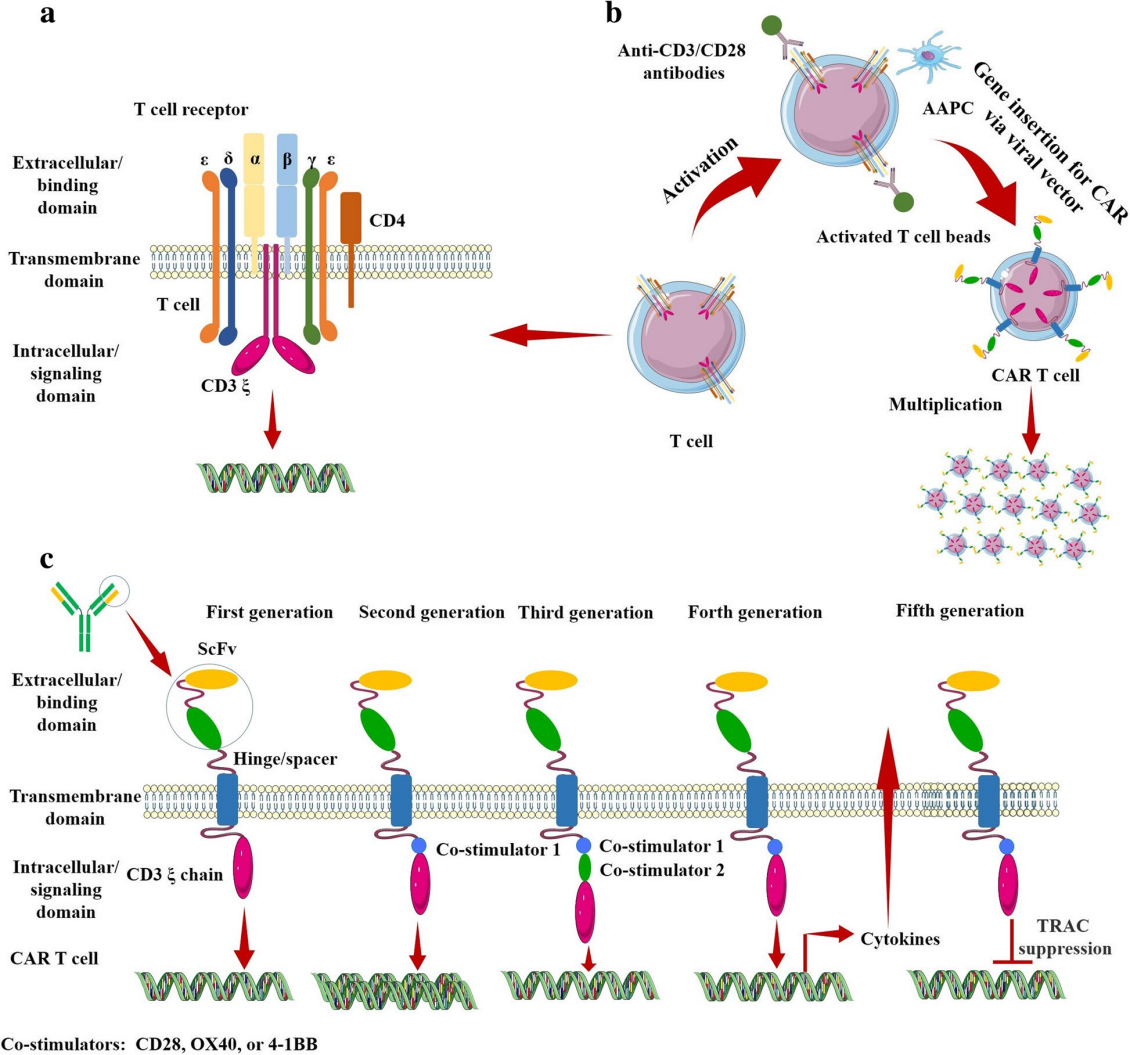
Structural features of T cell receptor versus CAR T cell design. **a** Structural features of T cell receptor. **b** Production of CAR T cells from patient-derived T cells. **c** Structural features of CAR T cell design of different generations. The principal CAR structure includes an extracellular/binding domain, a transmembrane domain, and an intracellular/signaling domain. The extracellular portion of CAR is typically generated from a monoclonal antibody against the target, which is also known as a single-chain variable fragment (ScFv). The ScFv is affixed to the transmembrane domain that crosses the cell membrane via the hinge/spacer region. Following the recognition and binding of ScFv part of the CAR with tumor antigen, the intracellular/signaling domain comprised of co-stimulators and the CD3ζ chain initiates intracellular signaling. The first-generation CAR contains only immunoreceptor tyrosine-based activation motif (ITAM) motifs in the intracellular domain. The second-generation CAR includes one co-stimulatory molecule, and the third-generation CAR contains two co-stimulatory molecules. The fourth and fifth generations of CARs are based on second-generation CAR. The fourth-generation CAR contains 1–3 immunoreceptor tyrosine-based activation motifs combined with an inducible expressed cytokine; while the fifth-generation CAR is T cell receptor-deficient CAR T cells developed employing genome editing technologies

CAR T cells exhibit immunotherapeutic promise by utilizing the remarkable specificity of the antibody(ies) and/or target ligand(s) to guide the cytolytic ability of T cells. CARs mimic the canonical T cell pathway by virtue of the inclusion of either a single-chain variable fragment (ScFv) derived from a monoclonal antibody or a mutated ligand for target binding, one or more co-stimulatory domains, and the ξ chain of the CD3 complex within a single multi-domain receptor (Fig. 1) [13]. The introduction of CAR into T cells allows them to eliminate cells expressing specific target antigen(s) through the same effector functions utilized by wild-type T cells to kill infected or transformed cells (Fig. 1). CAR T cells can broadly be grouped into five generations based on how their intracellular signaling domains are organized, despite the fact that their essential modular structure has not changed since their inception (Fig. 1). The CD3ζ alone makes up the first generation of CARs, while additional co-stimulatory signaling domains are present in the second generation [14]. Two co-stimulatory domains are combined in the third generation. TRUCKs i.e. T cells redirected for antigen-unrestricted cytokine-initiated killing are fourth-generation CAR T cells that express chemokines such as (IL)-12 when activated. Compared to earlier generations, the fifth generation of CAR T cells contains an additional intracellular domain [15]. These CARs consist of truncated intracellular domains of cytokine receptors with a transcription factor-binding motif. The TRAC gene is inactivated in the fifth generation of CARs by virtue of gene editing methods, resulting in the removal of the T cell receptor α (TCR-α) and β (TCR-β) chains. Overexpression of CARs on endogenous T cells boosts their ability to target and kill specific cells.

They even present with unique advantages of long residence time and proliferative abilities, making them comparable to ‘living drugs’ [16]. Certain chemokines and adhesion molecules crucial for T cell trafficking into the brain have been identified. The immunosuppressive microenvironment is shaped by mutations in the isocitrate dehydrogenase (IDH) genes IDH1 and IDH2 in glioma cells, which inhibit STAT1 expression and decrease the production of CD8 T cells, type 1 related effector molecules, and chemokines e.g. CXCL10 [17]. In line with the findings, T cell infiltration was significantly lower in IDH-mutant gliomas compared to IDH-wildtype gliomas [18]. Clearly, identifying and incorporating mechanisms that induce T cell surveillance and trafficking, into CAR T design might improve the systemic antitumor response against solid brain tumors. Stress responses arising from a lack of essential amino acids in the brain tumor microenvironment impact T cell functions negatively. Tumor-associated myeloid cells, assisted by regulatory T cells often encourage the production of immunosuppressive agents like ILs, especially IL-4 and IL-10, arginase 1, indoleamine 2,3-dioxygenase (IDO), etc. in the tumor microenvironment. Thus, CAR T therapy holds beneficial promises from the selective targeting of immunosuppressive myeloid cells in the tumor microenvironment. The therapeutic potential of CAR T cells would be significantly increased by introducing immunosuppressive resistance into the cells themselves, in combination with additional drugs that promote T cell activity.

Understanding the complexity of tumor heterogeneity is crucial in order to increase the repertoire of antigens for CAR T cells and find optimal targets to put an end to brain tumor antigen escape. Studies have revealed that the leading edge of tumors exhibits a proneural-like characteristic, whereas the tumor core poses with a more mesenchymal-like appearance [19]. It is also interesting to note that single-cell RNA sequencing has revealed a non-autonomous regulatory mechanism for brain tumor heterogeneity [20]. Recurrent glioblastomas are known to demonstrate reduced monocytes compared to primary glioblastoma. Advancing CAR therapy against brain tumors requires strategies to minimize antigen escape caused by tumor heterogeneity.

## Clinical successes of CAR T in hematological cancers igniting the hope

As a result of the impressive clinical success achieved by CD19-specific CAR T cells in the field of hematological malignancies, the United States Food and Drug Administration (FDA) has approved some CAR T cell-based therapeutics against leukemia, multiple myeloma, and lymphoma (Table 1) [21, 22]. While T cells require both the shape of the T cell receptor and the presentation of the MHCs in order to recognize tumor-associated antigens produced by cancer cells, CAR T cells exclusively rely on the CAR structure. Both the antigen-binding specificity of CARs and the cytotoxicity of T cells are present in CAR T cells. CD19 is used most frequently to treat hematological cancers. When injected into the body, CD19 CAR T cells target all CD19-positive cells. Through extensive proliferation and phosphorylation, CAR T cells start the activation process. Cytotoxicity and the release of cytokines are the major anticancer response mechanisms. CD8+ CAR T cells are crucial to the process of eliminating tumor cells. CD4+ CAR T cells provide a supporting role that can improve the immune response against tumors. Granzyme and perforin, which are capable of harming tumor cells, are secreted by CAR T cells to exert cytotoxicity [23]. The other method of cytotoxicity involves inducing apoptosis in cancer cells by endorsing apoptotic signal transduction. CAR T cell-released cytokines improve tumor clearance by stimulating a variety of immune cells and producing synergistic effects.

**Table 1:** FDA-approved, CAR T-mediated therapies for hematological cancer

S No	Trade Names	Proper Names	Route of administration	Indicated diseases	Years of approval
1	KYMRIAH	Tisagenlecleucel	Intravenous infusion	Lymphoma	2017
2	YESCARTA	Axicabtagene ciloleucel	Intravenous infusion	Lymphoma	2017
3	TECARTUS	Brexucabtagene autoleucel	Intravenous infusion	Lymphoma, acute lymphoblastic leukemia	2020
4	ABECMA	Idecabtagene vicleucel	Intravenous infusion	Multiple myeloma	2021
5	BREYANZI	Lisocabtagene maraleucel	Intravenous infusion	B cell lymphoma, follicular lymphoma	2021
6	CARVYKTI	Ciltacabtagene autoleucel	Intravenous infusion	Multiple myeloma	2022

CAR T cell therapy is undeniably a promising tool for future cancer immunotherapy. Longer persistence of CAR T cells has been demonstrated to improve anti-cancer response to hematological cancers, which seems to be replicated for CAR-mediated treatment of other types of cancers too [24]. In many cases, brain malignancies share common targetable tumor antigens and peptides with hematological cancers e.g. B7-H3, CD70, etc. [25]. Moreover, CD19 and/or CD28-based CAR moieties with co-stimulatory domains for 4-1BB seem equally promising for both hematological and brain cancers [26]. The fact that CAR T cells, modified immune cells from the patient body, are not constrained by CNS barriers fuels more optimism for a cure for brain cancer.

## CAR T cells for brain tumors

Following promising clinical outcomes with CD19-CAR T therapy against hematological cancers, the implication of CAR T cell-based therapeutic approaches for the treatment of solid tumors, including brain tumor has sparked huge interest among the scientific community. After treatment, analysis of the cerebrospinal fluid (CSF) revealed that the systemically administered CAR T cells frequently travelled to the CNS [27, 28]. Due to factors such as the distinct tumor microenvironment, the challenge of accessing the tumor(s), and heterogeneity in the expression of the target antigen, CAR T cells initially failed to replicate their effectiveness against hematological cancers in the brain. The subsequent generations of CAR T cells have been prepared in an effort to get beyond the obstacles faced by initial CAR T cell technologies in the treatment of brain cancers. Rapid developments in gene editing technology have opened up a number of possibilities for CAR T cell modification e.g. cytokine overexpression, gene knock-out and knock-in, simultaneous targeting of multiple antigens, precise control of CAR expression and signaling to increase their effectiveness [29]. The suppression or improper presentation of tumor antigens, which occur frequently in brain tumors due to abnormalities in components of human leukocyte antigen (HLA) class I antigen processing machinery has no adverse effects on this form of therapy because recognition and effector mechanisms of CAR T cells are HLA class I independent. In addition, CARs supersede T cells in that, besides small peptide sequences, they can also recognize tumor antigens in the form of carbohydrates, glycolipids, and proteins [30].

### Design considerations

The optimization of CAR design is highly dependent upon antigen and tumor-specific properties. At the first step of designing CAR T cells, patient T cells are collected by leukapheresis and a specific subset of T cells are collected. T cells are activated by engaging their T cell receptors, and co-stimulatory receptors (mostly anti-CD3 and anti-CD28) and/or adhesion molecule(s) (Fig. 1). Stimulated T cells are genetically modified by viral transduction to intensify the expression of CAR. The engineered CAR T cells are expanded ex vivo in media supplemented with common γ-chain cytokine cocktails e.g. IL-2, IL-15, IL-7. And IL-21. Superior CAR T cell products are generated by a less differentiated memory phenotype with increased mitochondrial fitness [31]. Ex vivo cultures of about 2 weeks are capable of producing millions of engineered CAR T cells with therapeutic potential to be infused back into the patient. Certain factors, such as the effect of intrinsic T cell product variability on efficacy, consistency over time, and the extent of T cells reaching the CNS, need to be taken into consideration when using CAR T cells to treat brain tumors.

CAR T cells have been designed to secrete proinflammatory cytokines to aid in their function and proliferation while serving as a defense against immunosuppressive cytokines. In solid tumor models, CAR T cells secreting IL-12, and IL-18 have been evinced to exert longer-lasting tumor responses preclinically [32, 33]. Enhanced anti-tumor efficacy of CAR T cells with constitutive IL-7 and IL-15 signaling has also been reported, as well as with the inducible release of an IL-15 super-agonist complex by T cells upon interaction with the cognate antigen [34, 35]. Secreted pro-inflammatory molecules, in addition, exert some paracrine effects like reshaping the tumor microenvironment to be antitumorigenic, and regression of solid tumors [36, 37]. Co-expression of dominant-negative transforming growth factor β (TGFβ) receptor II (TGFβRII) can block TGFβ signaling within CAR T cells leading to reduced exhaustion, and improved anti-tumor efficacy [38]. As a measure of imparting resistance to the immunosuppressive tumor microenvironment, engineered T cells can be made to recognize soluble ligands so that the immunosuppressive cytokine signal can potentially be converted into an immune-stimulatory one [39, 40]. CAR T cells engineered to secrete a programmed cell death ligand 1 (PD-L1) antibody, CAR T cells with programmed cell death 1 (PD-1) and lymphocyte activation gene 3 (Lag3) genes knocked out using clustered regularly interspaced short palindromic repeats (CRISPR)/ CRISPR-associated protein 9 (Cas9) technology, and CAR T cells designed with a PD-1 ectodomain linked to the transmembrane, and cytoplasmic domains of CD28 in order to convert an immunosuppressive signal into a co-stimulatory one have all been explored exhibiting encouraging outcomes [41–43].

**Fig. 2:**
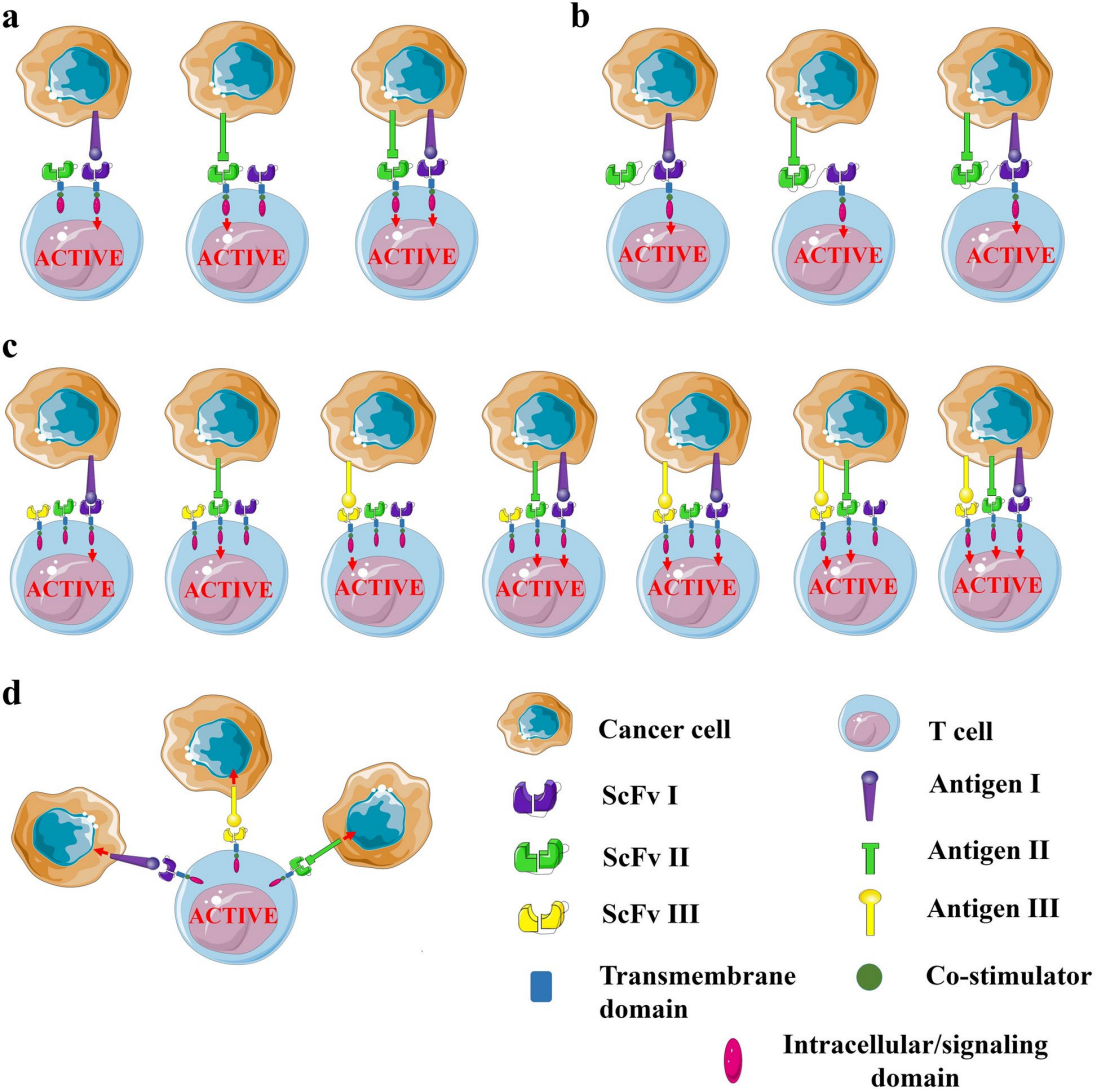
Multi-antigen targeting CAR T cells that prevent tumor antigen escape to intensify therapeutic attributes. **a** Bispecific/bivalent ‘OR-gated’ CAR T cells. Each CAR exhibits a comprehensive signal domain that, when present with either alike antigen, intensifies the antitumor activity of CAR T cells. **b** Bispecific ‘tandem’ CAR T cells. One CAR simultaneously expresses two different antigen-binding domains. **c** Trivalent ‘OR-gated’ CAR T cells. Three CARs expressing T cells intensify the antitumor effects by recognition of one, two, or three tumor-specific (targeted/validated) antigens. **d** A mixture of different single-targeted CAR T cells also ensures multi-antigen targeting

Tumor antigen expression on normal tissues often hinders the usage of CAR T cells due to the concern of unexpected side effects and toxicities in healthy tissues. To overcome this challenge CARs designed with ScFvs with varying affinity allow for differential recognition of the targeted tumor antigen [44]. Despite several developments, recurring tumors might still circumvent monovalent CAR T cells by downregulating the targeted antigen or by creating antigen-negative clones [45]. Encompassing bispecific target domains within CAR design can minimize antigen escape by rendering recognition of either of two targeted antigens for activation of T cells (Figs. 2a and b). Well-characterized antigens remain the focus for designing bispecific ‘OR-gated’ CARs against brain tumors (Fig. 2a). This strategy can be highly useful in the case of recurrent tumors where malignant cells devoid of the single targeted antigen have been expressed as a result of earlier CAR T treatment. In a CAR-based study, bispecific targeting of IL-13 receptor α2 (IL13Rα2) and human epidermal growth factor receptor 2 (HER2) was able to control glioblastoma for nearly a month whereas single-targeted CAR therapy was ineffective due to antigen escape [46]. Regarding design considerations of bispecific CARs, the ‘tandem’ design sequentially arranges the heavy and light chains of each ScFv while the ‘loop’ pattern resembles the bivalent antibody structure (Fig. 2b). In a study with CD19/CD22 bispecific CARs, the ‘loop’ design proved overwhelmingly superior to the ‘tandem’ design stressing the importance of bivalent designs, linker lengths, and order of ScFv for CAR performance [47]. The incorporation of a third targeting domain (trispecific CARS) can further intensify this approach (Fig. 2c) [48]. Another exciting approach involves the use of EGFRvIII-specific CAR T cells engineered to secrete bispecific T cell engagers (BiTE) [49]. These bispecific monoclonal antibodies can link T cells to wild-type EGFR, overcoming the resistance of EGFRvIII heterogenous glioblastoma to EGFRvIII-specific CAR T cells. Alternatively, multi-antigen targeting can be addressed by expressing two CARs on the same T cell, and/or by mixing different single-targeted CAR T cells (Fig. 2d) [50, 51]. Interestingly, monitoring of safety and efficacy of mixed CAR T cells might be easier from previous experience with single-targeted CAR T cells. On the contrary, bispecific CAR T cells seem to be more effective experimentally compared to a pool of single-targeted CAR T cells, probably because of the effects of local competition [46, 52]. The future of CAR T cell engineering lies in overcoming reliance on selective tumor antigen(s) and allowing them the flexibility to target several antigens, while at the same time imparting in them the capacity to be turned on or off in a timely and effective manner to avoid toxicities. Thus, when designing new approaches, safety switches such as suicide genes, and using inducible and/or controllable CAR systems are attractive approaches to address the issue of CAR T cell-induced toxicity. SynNotch system utilizes ‘AND’ and ‘NOT’ gated CARs i.e. CAR expression can only be induced upon recognition of a second tumor-associated antigen [53]. The limitation of such an approach is that both antigens must be expressed at considerably high levels by tumor cells. This requirement poses a potential problem for anti-glioma CAR T cells due to high heterogeneity in antigen expression levels. Universal CARs have been designed in order to fight tumor heterogeneity by utilizing adapters to connect CAR with targeted antigens thereby permitting antigen switch without re-engineering T cells [54, 55]. Additional approaches to enhance CAR T cell anti-tumor activity include the incorporation of the hypoxia transcription factor-1 alpha (HIF-1α) subdomain in a CAR construct. This results in the activation of CAR T cells only under hypoxic conditions such as within a tumor microenvironment [56]. This strategy may attribute an on-target effect with minimized off-tumor toxicity.

### Delivery/trafficking

Brain tumors present unique challenges to host T cells, or so to say, any immune cells owing to the highly selective nature of the BBB and the blood CSF barrier (BCSFB). These barriers control immunological entrance into the brain and CSF and restrict immune extravasation through post-capillary vesicles. CAR T cells can be used as delivery vehicles when combined with constitutively or inducibly expressed cytokines, chemokines, antibody fragments, or other biomolecules. Circulating T lymphocytes must first attach to the vascular endothelium via integrins, adhesion molecules, and chemokines. In the absence of inflammation, activated T cells can be recruited beyond the BBB, however, CD8 T cells still require the presentation of the major MHC class I antigens on luminal endothelial cells.

Systemic administration of CAR T cells through the intravenous route is most common in cases of hematological and solid tumors. Experimentally, CD19-CAR T cells were shown to reach CNS and combat malignancy [27]. The adoptively transferred cells were activated in the periphery due to the presence of disease. The fact that activated T cells are more likely to travel to the CNS may have a considerable impact on the effectiveness of CNS trafficking. Locoregional routes including intratumoral, and intraventricular routes of administration bypass most of the barriers to reaching brain parenchyma. Comparative studies revealed that local administration of CAR T cells beats systemic delivery for glioblastoma, and breast cancer brain metastases, with improved targeting of multifocal disease, thus illustrating the surveillance of CAR T cells throughout the CNS [57–59]. From a safety point of view, locoregional distribution is anticipated to reduce off-tumor targeting of other systemic tissues and intravenous first-pass pulmonary damage [60].

CAR T cells can be engineered to express chemokine receptors to enhance intra-tumoral T cell trafficking. A clinical trial has revealed that CD19-CAR T cells can fight CNS leukemia by trafficking into the CNS [27]. Interestingly, the forced expression of C-C chemokine receptor type 4 (CCR4) could enhance CAR T cell accumulation and therapeutic response in Hodgkin’s lymphoma [61]. Detection of chemokine (C-C motif) ligand (CCL)2, CCL4, CCL5, CCL17, and CCL21 which have all been linked to glioma also, ignite promise regarding the efficacy of them against glioma. However, CCR4 may be a flexible tactic for brain cancer [62, 63]. Tumor tropism of adoptively transplanted T lymphocytes by controlling tumor chemokine release to glioblastoma expressing CCL2, (a ligand for CCR4) has been demonstrated [64, 65]. In line with the findings, the incorporation of CCR2, a receptor for CCL2 in CAR T cells has enhanced efficacy against neuroblastoma [66]. Chemokine receptor-engineered T cells should be designed for the chemokine signaling specific to each tumor type to improve desired localization. Additional targeting of endothelial adhesion molecules or vascular cytokines (upregulated in brain tumors) could also be a viable strategy for increasing CAR T cell accumulation at the tumor site [67, 68].

Using imaging techniques to track the migration of CAR T cells in real time can offer significant evidence for on- and off-target localization, compared to the sampling of blood, CSF, or invasive biopsies, which provide more generalized information about cells in circulation or in the biopsied tissue, respectively. Nuclear imaging techniques (single-photon emission computerized tomography, positron emission tomography, etc.), coupled with anatomical detailing (computerized tomography scan, magnetic resonance imaging, etc.) can enable sensitive, non-invasive tracking of perfusion in brain tumors [69, 70]. Several reporter systems using human genes as non-immunogenic alternatives are being tested with the aim to study the trafficking of T cells while simultaneously minimizing the risk of immunological rejection. Though this strategy yields signals based on the extent of the presence of metabolically active cells, it is also influenced by background uptake of the nucleoside, and the BBB permeability and tissue penetrability of the probe [71, 72]. Cell-antigen-specific visualization with positron emission tomography has been applied to track T cells engineered with antigen tags. Metal chelator-based strategies are also displaying promise, especially in tracking CARs with ScFvs [73]. In summary, pre-labelling techniques should be employed when sensitivity and early trafficking are crucial, while injected antibodies and reporter genes perform better over repeated imaging sessions and the signal is connected with the number of living cells.

## Clinical considerations

Brain cancer is possibly the current forerunner among different tumor types to undergo clinical trials with CAR T cell-based treatments. Multiple potential targets for CAR T cell-based therapies have been identified through immuno-histochemical investigations of brain malignancies. In the majority of cases, malignant brain tumor patients in advanced stages are enrolled after verification of expression of targeted tumor antigen(s). The intended dose is divided into multiple weekly infusions. Splitting of doses minimizes the safety risks compared to single bolus administration [74]. In addition, repeated administration allows increased overall dosing of functional CAR T cells over a longer duration of time, thus lengthening the therapeutic window. Positron emission tomography and magnetic resonance imaging are undertaken to evaluate the response of CAR T cells in volunteers/patients. Immunologic correlative studies are conducted over the course of treatment to assess the persistence of CAR T cells in peripheral blood and CSF. Continuous monitoring for activation of the endogenous immune system indicated by changes in cytokine levels in blood and CSF is also undertaken simultaneously. Alterations in antigen expressions, and variations in cytokine levels in the tumor milieu are also monitored.

### Targets attributed clinical promise

**Fig. 3:**
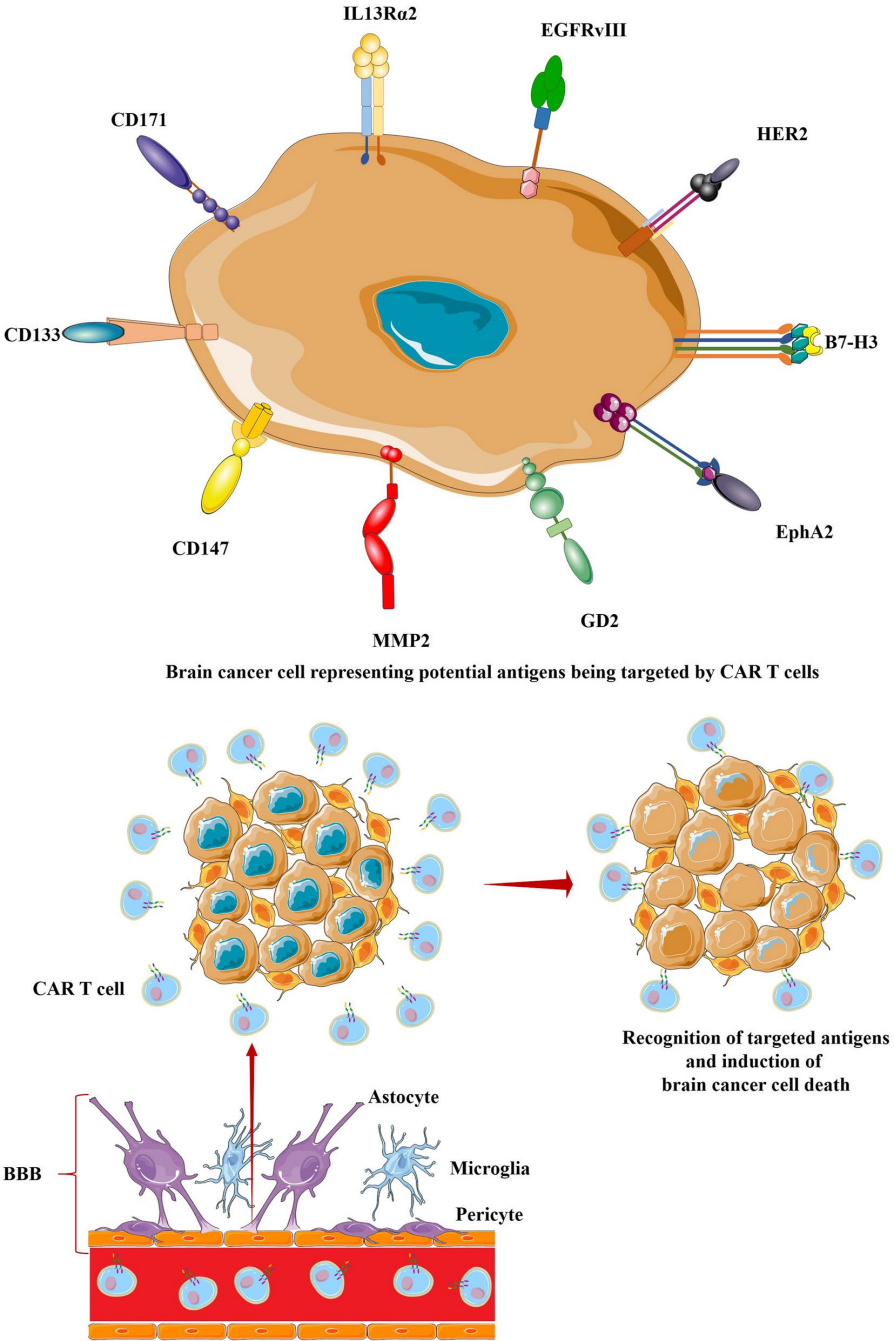
A glance at CAR T cell-based therapeutic prospect in brain cancer management. CAR T cells recognize some brain tumor-specific antigens that are targeted with an ambition to eliminate brain cancer cells. Some malignant growths in the brain can damage the BBB and help CAR T cells to cross the BBB to reach the tumor site. In addition, some delivery strategies allow delivering CAR T cells to the tumor site enabling the achievement of better therapeutic efficacy in brain tumor management

Targeting a small number of antigens, the initial clinical trials with CAR T cells for brain tumors have started with most patients with recurrent or resistant glioblastoma. Lead tumor antigen candidates were chosen based on evidence of negligible expression in the normal brain, and the history of overexpression on malignant cells (Fig. 3). This is particularly important to reduce off-target toxicities. Target-based evaluation of CAR T cell therapy for brain cancer management is uncovering salient insights regarding the safety and bioactivity of the same, steering toward future clinical translations.

#### IL13Rα2

IL13Rα2, a high-affinity IL-13 receptor that is typically overexpressed on glioblastoma cells and barely expressed on healthy brain tissue, was one of the first targets for CAR T cell therapy of brain tumors. IL-13 can bind to the decoy receptor IL13Rα2, which lacks a functional cytoplasmic domain, thus unable to trigger an intracellular signaling pathway [75]. Expression of IL13Rα2 increases with advancing stages of the disease. The mesenchymal subclass of glioblastoma is also associated with IL13Rα2 related gene signatures [76]. Importantly, both glioma stem-like cells and differentiated tumor cells express IL13Rα2, thus maximizing the circumference of IL13Rα2-targeted CAR T cell therapy.

An E13Y site-directed mutation was introduced in IL-13 based on work on cytotoxin-conjugated IL-13 that defined mutations increasing the specificity of IL-13 for IL13Rα2 over the more ubiquitously expressed IL13R1/IL4R complex [4, 77]. The engineered CAR T cells acted specifically upon glioma cells consistent with the expectations.

Second-generation CARs designed with an additional 4-1BB co-stimulatory domain and optimized spacer domain, presented a nearly 10-fold increase in anti-tumor potential compared to first-generation IL-13 zetakines [58, 78]. During the initial days, two clinical trials were undertaken to evaluate the feasibility and safety of locoregional intracranial tumoral delivery of autologous [79], and allogenic [80] CAR T cells (first-generation IL-13 zetakine CD8+ T cell clones) for resectable, and non-resectable glioblastoma, respectively. While short-lived anti-glioma activity was observed among a few patient volunteers, dose-limiting toxicity was completely absent at a dose of 1 × 10^8^ CAR T cells. The first-generation IL-13 zetakine CAR T cells demonstrated limited persistence, in line with the transiency of the anti-glioma effect observed and led to initiatives to improve CAR design for the betterment of therapeutic efficacy [57, 81]. Second-generation IL13Rα2-targeted, 4-1BB co-stimulatory CAR T cells underwent clinical trial [82] whereby CAR T cells were introduced in r/r IL13Rα2+ malignant glioma patients through either intratumoral, intraventricular or dual intratumoral/intraventricular routes. With the phase I study still ongoing, safety and tolerability with regard to the absence of dose-limiting toxicity are already reported [57]. One of the patients has experienced dramatic upgradation regarding the quality of life, including discontinuation of glucocorticoids on receiving intraventricular CAR T cell administrations that lasted for about 7.5 months. Increases in endogenous immune cells and inflammatory cytokines detected following each intraventricular infusion of CAR T cells may be due to the activation of the host immune system. Thus, a complete response was generated inspite of non-uniform expressions of IL13Rα2 on respondent tumors. Recurrent tumors appeared outside the brain with lowered expression of IL13Rα2 highlighting antigen escape as a mode of tumor resistance to immunotherapy. Another trial [83] is still assessing the effect of IL13Rα2 CAR T cells on medulloblastoma, glioblastoma, and ependymoma. CAR T cells after lymphodepletion for the treatment of IL13Rα2 positive recurrent or refractory brain tumors in children are under clinical evaluation [84].

#### EGFR

EGFRvIII, a deletion mutant of endogenous EGFR, frequently exhibits genetic amplification and/or mutation during glioblastoma, it is an attractive target for CAR T treatment. EGFRvIII presents multiple advantages viz. tumor-restricted expression of mutation, and an immunogenic epitope in the form of a truncated extracellular domain responsible for activation of signaling cascades for the onset and progression of glioblastoma. The tumor-restricted expression profile of EGFRvIII makes it an appealing target. However, this receptor’s expression is unstable over the course of the disease, raising the possibility of antigen-negative escape variants under targeted therapy. Targeting wild-type EGFR could not solve the issue completely as the expression of wild-type EGFR is not restricted to tumor cells only, thus triggering the likelihood of off-target toxicity [85].

In a clinical trial [86], recurrent glioblastoma patients were treated with third-generation, ScFv-based EGFRvIII-targeted CAR containing co-stimulatory domains for CD28 and 4-1BB. Intravenous administration of CAR started with doses as low as 1 × 10^7^ cells followed by systemic delivery of IL-2. At low doses of 1 × 10^7^ to 1 × 10^9^ cells, no evidence of off-tumor toxicity was observed. However, higher doses of ≥1 × 10^10^ cells, pulmonary toxicities became prevalent. At a dose of 3 × 10^7^ cells, one patient encountered hypoxia and dyspnea as serious adverse effects. One death was recorded at a dose of 6 × 10^10^ cells whereby pulmonary edema was evidenced on post-mortem examination [87]. These unwanted effects might be attributed to activated T cells congesting the pulmonary vasculature in a dose-dependent manner, which is probably a limitation of the route of administration used. It is worth mentioning that EGFRs are expressed in pulmonary cells too. Overexpression of EGFRvIII on lung carcinomas has been utilized for CAR targeting whereby EGFRvIII-targeted CAR vectors have demonstrated promising anti-cancer activity against non-small cell lung carcinoma both in vivo and in vitro [88]. In the clinical trial under discussion [86]. CAR T cell persistence was on the lower side resulting in median survival of 6.9 months. Despite the fact that the findings primarily emphasize the difficulties in using CARs to treat brain tumors, one patient gave rise to some optimism by managing 12.5 months of progression-free survival. A humanized EGFRvIII-specific CAR exhibited promising activity and safety profile against glioblastoma in preclinical studies [89]. Thus, it was advanced to a phase I clinical evaluation [90]. Patient volunteers received a single intravenous infusion of the engineered CAR vectors followed by surgical resection. Significant movement of CAR T cells to the desired site triggered optimistic promise. Downregulated EGFRvIII in recurrent tumors indicated initial antigen-specific targeting by CARs. Due to the inceptive heterogeneous expression of EGFRvIII on glioblastoma, the designed CARs could only target a fraction of malignant cells. The immunosuppressive tumor microenvironment was exacerbated by CAR T cell administration, including increases in indoleamine 2,3-dioxygenase (IDO1), programmed death-ligand 1 (PDL1), and IL-10. Non-CAR T cells, primarily polyclonal regulatory T cells, also contributed to the immunosuppression by virtue of their expression of CD4, CD25, and forkhead box P3 (FoxP3), which are markers for regulatory T cells [91]. The immunosuppressive response to CAR T treatment raises the possibility that protective measures like immune checkpoint inhibition can complement EGFRvIII-CAR T therapy, thus igniting possibilities of combination therapy. Further digging into this idea, another clinical trial [92] evaluated the synergistic response of EGFRvIII-CAR T cells with the anti-PD-1 antibody pembrolizumab, which has been discussed later on. Clearly, low affinity to endogenous EGFR and enhanced binding to tumor-specific EGFRvIII binding is necessary for the high efficacy of CARs. Antibodies such as mAb806 have been designed to target activated EGFRvIII and amplified EGFR. A clinical study with an indium-EGFR806 antibody has displayed nearly zero uptakes by normal tissues [93]. Clinical trials are on the way [94, 95] at Seattle Children’s Hospital. USA with EGFR806-CAR to assess on target-off tumor behavior on pediatric recurrent and/or refractory solid tumors, especially within the CNS.

#### HER2

HER2 is a highly alluring target antigen owing to its high abundance in brain tumors, its role in tumor progression, and the capacity of HER2-specific CAR T cells to eradicate both differentiated cells and cancer-initiating cells [96]. However, in the clinical setup, the first patient treated with HER2-targeted CAR T therapy succumbed to death, raising safety concerns [97]. Intravenous infusion of a dose of as high as 1 × 10^10^ cells led to death led to off-target toxicity on lung epithelial tissue, triggering cytokine storm, respiratory distress, and pulmonary edema as a result of the accumulation of HER2-CAR T cells in the lung and abdominal/mediastinal lymph nodes. Failure of the ScFv from high-affinity trastuzumab antibody, and CAR co-stimulatory domains consisting of both CD28 and 4-1BB mandated scientists to restructure the design. A newer optimized CAR containing ScFv from lower affinity trastuzumab, redesigned co-stimulatory domains lower cytokine release, and use of lower affinity HER2-monoclonal antibody FRP5 for improved tumor targeting advanced to investigative stages [98]. Initially, these HER2-CAR T cells demonstrated a satisfactory safety profile with low persistence in sarcoma patients [99]. The CARs were engineered into virus-specific T cells in an attempt to enhance their persistence assuming co-stimulation would result from the engagement of T cells with latent virus antigens on antigen-presenting cells. In a study on 16 patients [100] with progressive glioblastoma, the safety of autologous HER2-CAR virus-specific T cells was instituted. Though these CAR vectors established safety, their efficacy was below par highlighting the requirement for improvements regarding function, persistence, and expansion. In glioblastoma patients, up to 1 × 10^8^ HER2-specific CAR T cells with a CD28ζ endodomain were successfully administered without dose-limiting toxic effects [101]. Inspired by preclinical outcomes of low cytokine production of good anti-tumor efficacy of 4-1BB versus CD28 co-stimulation, two phase I clinical trials [102, 103] have started with optimized HER2-CARs among patients with HER2-positive malignant glioma and brain metastasis of HER2-positive breast cancer, respectively [59]. Interestingly, HER2-amplified breast cancer depicts much higher expression of HER2 compared to glioma [104]. Thus, clinical trials under discussion might also shed light on the effect of locoregional intracranial ventricular delivery to increase specificity. Another two clinical trials [105, 106] are evaluating locoregional delivery of HER2-specific CAR T cells on HER2-positive, recurrent and/or refractory pediatric CNS tumors. Another interventional trial [107] has initiated in 2022 with HER2-specific CAR T vectors against child ependymoma.

#### Others

The reported success of CAR T therapy so far, emphasizes the necessity for an appropriate CAR target to be highly expressed across tumor(s) and intratumoral cellular subsets. Since brain tumor cells express multiple markers that are also shared by parts of the normal brain, it has been difficult to identify suitable antigens in brain tumors. Repercussions of off-tumor targeting are far less admissible within the CNS compared to other systems. Moreover, resistance to CAR T cells led by the heterogeneity of the tumor antigen(s) in brain tumors, rationalizes the necessity for a larger library of targetable antigens.

B7-homolog 3 (B7-H3) alias CD276, a member of the B7 family of immune checkpoint inhibitors, is expressed in cases of hematological cancers and solid tumors including higher grades of glioma. In most of the normal tissues, the immune-histochemical analysis does not detect B7-H3 since normal tissues lack the ability to translate B7-H3 mRNA due to hindrance caused by microRNAs (miRs). Apart from the expression on the tumor itself, negatively regulating T cell activation, B7-H3 is also expressed on tumor-associated angiogenic vessels and fibroblasts [108]. Thus, targeting of B7-H3 by CAR T cells can act by not only direct targeting but also by suppression of angiogenesis and by the disruption of the stroma. Gaining inspiration from the preclinical promise exhibited by Majzner et al. [109] and Tang et al. [110], a phase I/II clinical trial [111] has been initiated in 2022 to assess the effect of B7-H3 CAR T cell therapy in between two successive cycles of temozolomide in the treatment of recurrent and/or refractory glioblastoma. Another trial [112] is assessing B7-H3-Specific CAR T cell locoregional immunotherapy for diffuse intrinsic pontine glioma/diffuse midline glioma and recurrent or refractory pediatric CNS tumors.

CD147, also known as extracellular matrix metalloproteinase (MMP) inducer is a type I transmembrane adhesion molecule pertaining to the superfamily immunoglobulin. CD147 prompts the production of MMPs 1, 2, 3, 9, 14, and 15 from fibroblasts to degrade the extracellular matrix and aid in tumor progression. Interestingly, the extent of expression of CD147 on glioma cells inversely correlates with disease prognosis, thereby making it a potential CAR target during the early stages of the disease [113]. An early phase I trial [114] has attempted to shed light on the safety, tolerability, and efficacy of CD147-specific CAR T cells against recurrent glioblastoma.

The appearance of ganglioside GD2 in different tumor samples makes it a sought-after tumor antigen for CAR T-mediated targeting [115]. The report claimed GD2 acts as a cancer-initiating cell marker in the case of breast cancer, which further boosts its attraction as a target tumor antigen among researchers [116]. Orthotopic xenograft models of patient-derived diffuse midline glioma are efficiently eliminated by GD2-specific CAR T cells [117]. GD2-specific CAR T cells have been administered safely to neuroblastoma patients in earlier studies [118, 119]. In a clinical trial [120] with anti-GD2 CAR T cells against neuroblastoma, no dose-limiting toxicity was observed with 1 × 10^7^ cells/m^2^, along with undetectable CAR T cell levels in peripheral blood. Expansion of engineered vectors was observed at 1 × 10^8^ cells/m^2^ while disease progression restarted at day 45 when CAR T cells were no longer detectable. An interventional trial [121] is assessing the safety and efficacy of C7R (an IL-7 cytokine receptor) expressing GD2 CAR T cells in advanced stages of glioma. An interventional trial [122] is assessing anti-GD2 CAR T cells against GD2-positive, pediatric neuroblastoma. GD2 CAR T cells are also being assessed against diffuse pontine glioma and diffuse midline glioma, clinically [123].

Tumor-targeting peptides can be used as a tumor-targeting domain of CARs as an alternative to designs based on antibodies. Chlorotoxin (CLTX) is a small, nature-derived peptide able to bind to primary brain tumors while exhibiting negligible affinity for normal tissues [124]. On incorporation into CARs, CLTX reroutes T cells towards targeted tumor detection with little detectable off-target consequences. Earlier studies have also reported no dose-limiting toxicity in patients receiving CLTX-bioconjugates, thus adding fuel to the idea of utilizing it as a CAR target [125]. Moreover, CLTX-specific CAR T cells depicted promising anti-tumor effects in glioblastoma orthotropic xenograft models [126]. Interestingly, expressions of MMP2, chloride voltage-gated channel 3 (CLCN3), and annexin A3 (ANXA3) seem to be indispensable for the binding of CLTX to glioblastoma cells [96]. The bioactivity of CLTX CAR T cells significantly has been shown to be decreased when MMP2 was knocked out, demonstrating the requirement of MMP2 expression for efficient targeting. Gaining inspiration from the previous outcomes, a phase I clinical study [127] is presently evaluating the therapeutic effect of CLTX CAR T cells on patients with recurrent or progressive MMP2-positive glioblastoma.

Glioblastoma stem-like cells play a role in mediating resistance to radiation and chemotherapy. Thus, elimination of glioblastoma stem-like cells is a prerequisite for lowering tumor recurrence. In a novel approach to specifically target glioblastoma stem-like cells, CAR T vectors have been fabricated against CD133, one of the surface markers of glioblastoma stem-like cells. CD133 CAR T cells have displayed cytotoxic potential on patient-derived glioblastoma stem-like cells [128]; however, the safety aspect still persists as CD133 is also expressed on neuronal stem cells. CD171 is a neuronal cell adhesion molecule playing a vital role in adhesion, migration, and differentiation. It is also prominently expressed in treatment-resistant cancers. A phase I trial [129] is evaluating the safety and feasibility of CD171-specific CAR T cells against recurrent/refractory neuroblastoma.

It has been revealed that glioblastoma cells overexpress the erythropoietin-producing hepatocellular carcinoma A2 (EphA2) that plays an essential role in carcinogenesis and cell migration [130]. A clinical trial [131] had been initiated to examine the efficacy of EphA2-CAR T cells on glioblastoma patients but has been discontinued.

### Combination therapy

**Fig. 4:**
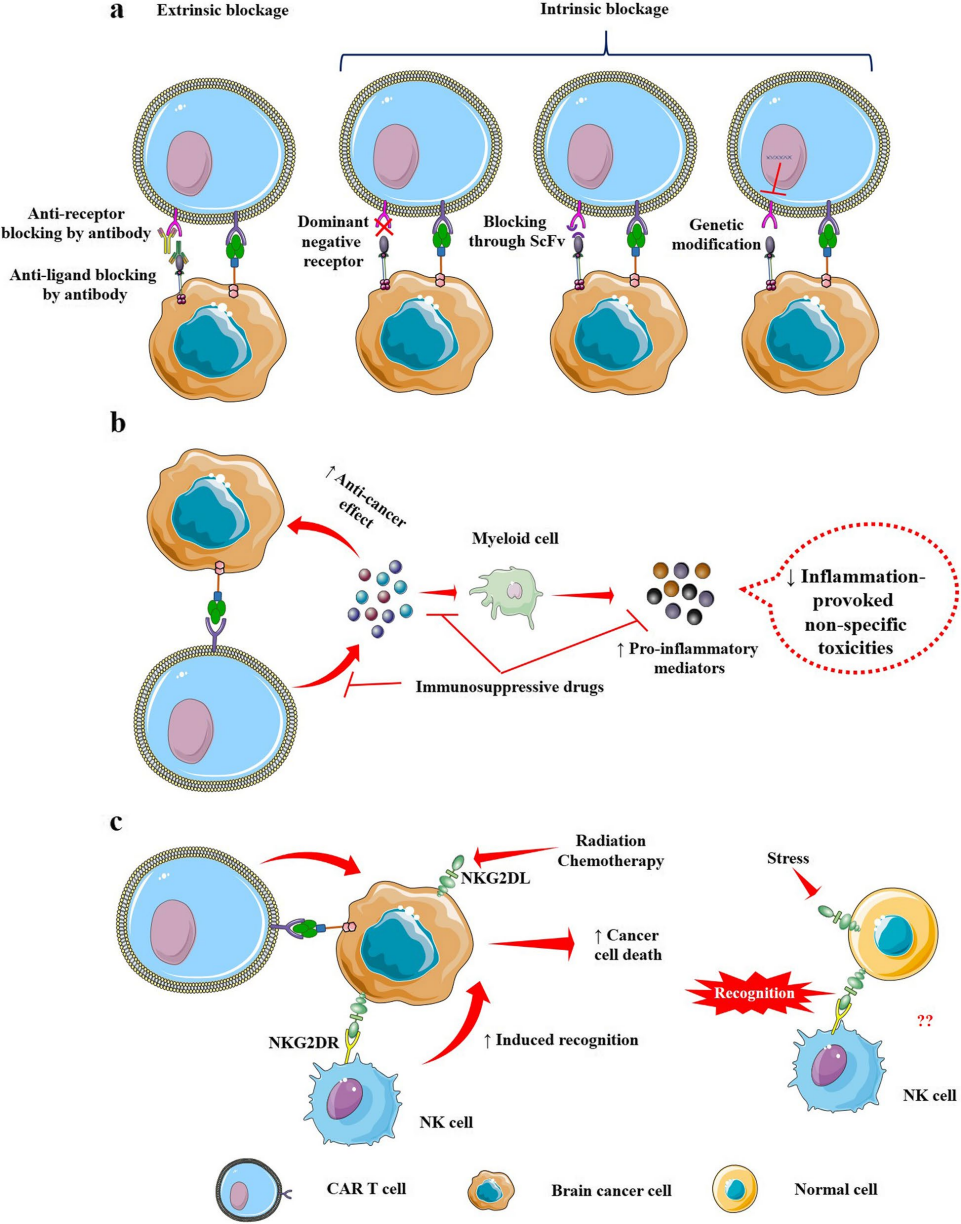
An overview of combination therapy with CAR T cells. **a** Combination of CAR T cells with checkpoint inhibitors. **b** Combination of CAR T cells with immunosuppressive drugs. **c** Natural killer group 2D (NKG2D) ligand-based targeting by natural killer cells coupled with CAR T cells highlights a potential for combinatorial treatment. Extrinsic checkpoint blockade rests on programmed cell death 1 (PD-1) receptor/ligand hindering antibodies. Genetic engineering enables intrinsic PD-1 checkpoint blockage to express proteins or nucleic acid that disrupt PD-1/PD-L1 signaling. The PD-1 dominant negative receptor, which competes with native PD-1 receptor and inhibits inhibitory signaling through native wild-type receptors, lacks the intracellular signaling domain in its creation. PD-1/PD-L1 inhibiting single-chain variable fragments (ScFvs), which provide local antibody inhibition of PD-1 receptor/ligand interaction, can also be ensured by CAR T cells. Furthermore, gene-editing techniques can eliminate PD-1 expression by editing the programmed cell death protein 1 gene locus. CAR T cell therapy coupled with immunosuppressants attenuates non-specific toxicities by suppressing inflammation. The usefulness of combination therapy in the treatment of cancer is reinforced by the fact that NKG2D ligand-based targeting of natural killer cells in conjunction with CAR T cells enhances the anticancer effect of CAR T cells

Combination therapy may be a prospective approach to address the unfavorable glioma environment and improve CAR T cell activity and durability. Combining CAR T cell therapy with a checkpoint inhibitor would aid in overcoming the barriers to T cell invasion and functionality. Two clinical studies have shown that neoadjuvant anti-PD1 immunotherapy can improve survival and modify the tumor microenvironment in glioma patients indicating blocking of PD-1 could alter the tumor microenvironment and act in conjunction with CAR T treatment to lengthen the survival of glioma patients [132, 133]. Alternative tactics might include genetically deleting PD1 from CAR T cell products or designing CAR T cells to produce a blocking antibody against PD1/PDL1. A combination trial of EGFRvIII-CAR T cells with an anti-PD-1 antibody (pembrolizumab) has been undertaken [92]. Another trial is evaluating the combination of IL13Rα2-CAR T cells with Nivolumab [134]. However, the safety, feasibility, and potential anti-tumor activity of CAR T cells in combination with checkpoint inhibitors are awaited from these trials. Another interventional trial [135] is assessing anti-GD2 CAR T cells along with IL-15, against GD2-positive, pediatric neuroblastoma. A schematic overview of checkpoint inhibition in combination with CAR T cell therapy has been depicted in Fig. 4a.

Drugs such as temozolomide, dexamethasone, and bevacizumab, which are often used in combination to manage and treat brain tumors, may have an effect on activation of CAR T cells, calling for careful monitoring. Although it is conceivable that lymphodepletion will enhance CAR T cells’ clinical responses against brain tumors, principally for intravenous administration, but its therapeutic efficacy in the case of brain tumors requires more clinical validation. The reported EGFRvIII-CAR clinical trials that included lymphodepletion preconditioning [86] did not show noticeably superior CAR T cell persistence, expansion, or patient outcomes when compared to a study that did not use lymphodepletion [87, 90, 91]. Though this could be due to a variety of factors, including CAR design, target selection, and patient enrollment, it emphasizes the importance of further clinical evaluation of the role of lymphodepletion in the application of CAR T cells for brain tumors, specifically for locoregional delivery. A dual-intensified regimen of temozolomide has been used as a preconditioning lymphodepleting regimen in an EGFRvIII CAR T [136] trial against grade IV glioma. As lymphodepletion has been proven to be crucial for the successful anti-tumor activity of CAR T cells regarding T cell receptors and tumor-initiating lymphocytes when the therapeutic cells are administered intravenously, the dual use of temozolomide for the treatment of glioma and for lymphodepleting preconditioning is an appealing method. On the other hand, dexamethasone. Frequently used to alleviate tumor-associated swelling of the brain, imparts toxicity to immune cells including T cells, and thus potentially apprehends CAR T cell activity. Dexamethasone has been demonstrated to impart deleterious effects in vaccine trials for glioblastoma at very low concentrations and is known to impede dendritic cell priming of the T cell immune response [137]. CAR persistence and effectiveness have been demonstrated to be disrupted by long-term systemic corticosteroid treatment in trials [74]. Given the significance of dexamethasone in the management of glioma symptoms, a successful CAR T cell trial design must identify its allowable concentrations. At a dexamethasone level of 4–6 mg/day, a clinically relevant response has been achieved with CAR T cells [57]. Bevacizumab, a non-steroid anti-inflammatory drug, has the potential to be an effective alternative to immunosuppressive corticosteroids like dexamethasone for treating cerebral edema symptoms [138]. Bevacizumab may also improve CAR T cell therapy by promoting tumor lymphocyte trafficking and counteracting the immune-suppressive effects of VEGF. Clinical validation of the benefits of combinatorial treatment of brain cancer with CAR T cells and bevacizumab is awaited, following promising preclinical outcomes [139]. An overview of the mechanistic impacts of different immunosuppressive drugs in combination with CAR T cells in the efficacious management of brain cancers has been depicted in Fig. 4b.

Stressed cells typically express natural killer group 2D (NKG2D) ligands, such as MHC class I-related chains and UL16-binding proteins, to help immune effector cells detect and eliminate them [140]. Glioblastoma cells, and glioblastoma stem-like cells both represent overexpressed NKG2D ligands. NKG2D ligand expression on glioblastoma cells can be increased by chemotherapy or radiotherapy, highlighting the potential for combinatorial treatment approaches [141]. Considering the expression of NKG2D ligands on normal tissues under stress, harm in humans cannot be completely ruled out. A phase I study [142] evaluating the clinical response of NKG2D-based CAR T vectors on solid tumors including glioblastoma, has been withdrawn citing administrative reasons. The mechanistic insight has been hypothesized in Fig. 4c.

## Preclinical advances

Resistance to CAR T cells is occurred due to the heterogeneity of the antigens presented by brain tumors, stressing the need for a larger library of targeted antigens. The effectiveness of CD19-CAR T cells highlights the requirement that a suitable CAR target is highly expressed across a variety of tumor types and intratumoral cellular subsets. The use of EGFRvIII-specific CAR T cells with disrupted PD-1 signaling via a CRISPR-Cas9 approach resulted in significantly longer survival of orthotopically engrafted mice [143]. Potential therapeutic use of B7-H3-CAR T cells against specific types of brain malignancies was shown by preclinical anti-tumor activity against a variety of pediatric tumors, including medulloblastoma [109]. In line with the finding, Tang et al. [110] also concluded that B7-H3 is commonly overexpressed in glioblastoma, and could be used as a therapeutic target in CAR T therapy.

Carbonic anhydrase (CA) IX is induced by hypoxia and is thus frequently overexpressed in many solid tumors, including glioblastoma. The extent of hypoxia in the tumor microenvironment regulates the level of CAIX expression in cancer cells, which is likely to lead to significant alterations in its expression in cancer cells [144]. CAIX-specific CAR T cells have been tested against glioblastoma cells in vitro and in vivo-xenografted mouse model after direct intratumoral injection [145]. Glioblastoma cells were successfully removed by CAIX-specific CAR T cells, thereby also enhancing the longevity of mice carrying tumors. However, CAIX expression in a patient might exhibit significant intra- and inter-tumor heterogeneity owing to the variability in the expression of CAIX on cancer cells depending on the degree of hypoxia. This expression pattern is likely to aid in the generation of cancer cell escape variants, which could pose a significant barrier to the successful application of CAIX-specific CAR T cell-based therapy.

CD70 is a transmembrane protein expressed on certain hematological and solid tumors besides being expressed on activated lymphocytes and matured dendritic cells. Human and mouse CD70-specific CAR T cells were able to recognize and eliminate CD70-positive glioblastoma tumors in vitro, as well as in xenograft and syngeneic models, without imparting significant toxicity [146]. CXCR1 and CXCR2-modified CD70-specific CAR T cells have been demonstrated to improve T cell trafficking and antitumor efficacy via IL-8-mediated chemotaxis in an in vivo glioblastoma model [147]. The higher CAR T cell antitumor activity resulted in improved tumor regression and survival of mice compared to those treated with unmodified CD70-specific CAR T cells. A major advantage of this tumor antigen is its confined expression mostly to malignant tumors [148]. Targeting CD70-positive T cells and dendritic cells may be of concern, but CD70-specific CAR T cells do not appear to have an impact on these activated immune cells [149]. In glioblastoma, preclinical investigations using CD70-specific CAR T cells have come up with promising findings, motivating researchers to move with this tumor antigen for possible clinical translations [150].

In gliomas, the expression of chondroitin sulfate proteoglycan 4 (CSPG4, also known as NG2) is inversely linked with patient survival [151]. Convincingly disproving the notion that CSPG4 expression on normal tissues might pose a problem when targeting this molecule as a tumor antigen, is the inability of CSPG4-specific CAR T cells to identify and lyse normal cells in vitro [152]. Further, in glioblastoma tissue and tumor-associated vasculature, CSPG4 is highly expressed with minimal heterogeneity but is not detected in the normal brain parenchyma [153]. In orthotopic a glioblastoma neurosphere xenograft model, intracranial injection of CSPG4 CAR T cells has been demonstrated to halt tumor progression [153]. In addition, the expression of CSPG4 on cancer-initiating cells is expected to allow CAR T cells to recognize and eliminate this particularly hostile cell subpopulation.

EphA2 is overexpressed in glioblastoma cells and glioblastoma cancer-initiating cells, without a detectable presence in normal tissues [154]. In vitro, EphA2 targeting CAR T cells effectively eliminates differentiated glioblastoma cells and glioblastoma cancer stem-like cells, significantly prolonging the survival of orthotopic xenograft mice [155]. A subsequent study utilized a short spacer region to fabricate an optimized version of the anti-EphA2 CAR construct [156]. Since the survival of glioma-bearing mice was prolonged to a similar amount utilizing a 20-fold lower dose, this CAR demonstrated stronger anti-glioma action compared to the previous CAR vector targeting EphA2.

Trophoblast cell surface antigen 2 (TROP2) is regarded as a stem cell marker and is expressed by solid tumor cells. High levels of this transmembrane glycoprotein have been demonstrated on surgically removed patient glioblastoma cells with minimal expression on normal brain tissues [157]. Since TROP2 is involved in the formation of new blood vessels in glioblastoma patients via upregulation of VEGF, targeting TROP2 might also aid to inhibit cancer growth by preventing neoangiogenesis. Encouraging outcomes with TROP2-specific CAR T cells against solid tumor cells viz. breast, pancreas, and prostate cancer cells is inspiring the scientific community to investigate similar vectors against glioblastoma cells [158].

Trispecific CAR T cells that target HER2, IL13Ra2, and EphA2 offer more thorough coverage of tumor antigens and have been demonstrated to considerably extend the longevity of mice, bearing glioblastoma patient-derived xenografts [48]. BiTE-armored CAR T cells successfully eradicated cancer cells and extended the survival of mice orthotopically grafted with either glioblastoma cells or patient-derived glioma neurospheres [49]. Engineering the CAR construct to induce or constitutively release active cytokines in order to boost CAR T cell activity and persistence is an approach to improve antitumor efficacy. In the orthotopic glioma xenograft mouse model, IL13Rα2 CAR T cells that were also designed to express IL-15 exhibited elevated anti-glioma activity, enhanced persistence, and considerably longer survival than the control [159].

Beyond membrane-associated proteins, the pool of brain tumor antigens that CAR T cells can target is anticipated to grow. Chheda et al. [160] revealed a shared neoantigen among patients with diffuse intrinsic pontine glioma as a result of a mutation in the H3.3K27 gene. When the altered peptide was used to stimulate HLA-A2+, and CD8+ T cells, the result was the creation of a TCR clone that, when expressed on T cells, caused cytotoxicity against tumor cells carrying the same mutation [160]. Additionally, CARs have also been created against soluble proteins, suggesting the possibility to target secreted components unique to brain tumors [40, 161].

## Current challenges with adoptive T cell therapy for brain cancer

CAR T treatment has not yet achieved the same level of resounding success against solid tumors as it has, in treating hematological cancers. Insufficient trafficking of CAR T cells to the tumor site, defective recognition of the targeted tumor antigen and expression of the targeted antigen on normal tissues result in off-tumor toxicity. Off-tumor toxicity may also be impacted by factors such as the limited durability and low proliferation of effector immune cells in the tumor microenvironment, and the uncontrolled expression and timing of effector activities that lead to deleterious effects and provide many escape routes. Several CAR T designing strategies have been undertaken to get rid of these challanges, which have been discussed in the earlier sections. With rare exceptions, CARs have the drawback of necessitating the expression of the target antigen on the cell surface, while T cell receptors recognize mostly intracellular moieties which are transported to the cell membrane by MHC class I antigens. Failure of CAR T cell treatments in glioblastoma may be caused by intratumoral heterogeneity, antigen/epitope loss after therapy, and selectivity. The ability of differentiated cancer cells to proliferate and endure, and the capacity of cancer-initiating cells to evade detection by CAR T cells owing to their lack of expression of the targeted antigen are thought to be the causes of the resistance. These cells also have the ability to advance the disease with a modified phenotype [162]. Thus, it is essential to select a tumor antigen with high homogenous expression and high-expression stability to inhibit this cancer escape mechanism. An ideal target is assumed to be expressed uniformly on all differentiated and cancer-initiating cells. Another alternative can be to develop strategies to improve the capacity of CAR T cells to destroy cancer cells that do not express the targeted antigen, may be by formulating bispecific or trispecific CAR T vectors. Cancer-initiating cells have been suggested to be partially responsible for treatment failure and disease recurrence due to their self-renewal ability and treatment resistance. CD133-specific CAR T cells have been shown to successfully eliminate glioblastoma cancer-initiating cells in an orthotopic in vivo model [128]. CAR T cells targeting B7-H3, CSPG4 and HER2 have also been shown to eliminate both differentiated glioblastoma and glioblastoma-initiating cells [153, 163].

Adhesion molecules on endothelial cells, as well as specific antigen expression by antigen-presenting cells, are thought to be required for the recruitment of antigen-specific CD8+ T cells across the BBB. T cell recruitment, on the other hand, is frequently reduced across BBB during CNS malignancies [164]. This decrease may give cancer cells an immune escape mechanism. Glioblastoma multiforme was earlier believed to uniformly damage the BBB; thus, drug, antibody, and immune cell permeability should not be an issue [165]. However, it has been demonstrated subsequently that, even in the presence of significant tumor burden, the BBB can remain intact [166]. To address this limitation, direct administration of CAR T cells to the tumor site has been investigated as a method of delivery, eliminating the need for cells to migrate across the BBB. Locoregional infusion of CAR T cells has been demonstrated to enhance T cell tumor infiltration and tumor control in brain malignancies as compared to intravenous delivery [58, 167, 168]. However, some concerns still persist regarding cytokine syndrome and neurotoxicity by the direct delivery of CAR T cells into the brain [169]. The incidence of high-grade neurotoxicity occurs in approximately 30% of patients treated with CD19-CARs [170, 171]. The gamut of these neurotoxicities is referred to as CAR T-related encephalopathy syndrome. CAR-related neurotoxicity is associated with disease burden, high CAR T dose, and cytokine release syndrome. CAR T cell-mediated CAR T-related encephalopathy syndrome was mimicked in a humanized mouse model with a high leukemia burden [172]. Since human monocytes were the primary source of IL-1 and IL-6 during CAR T-related encephalopathy syndrome, monocyte depletion alleviates toxicities. IL-1 blocking demonstrates a reduction of neurotoxicities; while, IL-6 receptor blockade can only inhibit the symptoms. The use of CAR T cells with suicide genes or switchable signaling components, multiple dosing of lower numbers of CAR T cells in a single infusion, choosing optimal CAR T cells with respect to binding avidity, co-administration of a steroid and/or a monoclonal antibody targeting proinflammatory cytokine receptors are the potential strategies that all have been investigated to reduce CAR T toxicities.

## Perspectives and critical analyses

Advancements in immunotherapy are igniting hope to treat solid tumors. As living T cell ‘micropharmacies’, CAR T cells can be used to deliver immunomodulatory molecules to the tumor microenvironment. Despite the majority of clinical trials are still in their early phases (Table 2), the results supported the safety of targeting certain tumor antigens in the CNS. Clinical trials using CAR T therapy [86, 90] have demonstrated progression-free survival for over a year; however, additional research is required to interpret the influence of underlying variations in tumor biology and the immunological microenvironment on the improved responsiveness of the patients. The inability of conventional preclinical models to accurately forecast the risk of toxicity in humans has been regarded as a significant challenge. In order to address these issues, various cutting-edge platforms that are currently available, such as suicide switches, RNA CARs, logic-gated CARs, etc. could be exploited.

**Table 2:** Clinical trials on CAR T-based management of brain cancer

S no.	Targets	Phases	Status	Indications	CAR designs	Administration routes	Comments	Identifiers
1	IL3Rα2	I	Completed	Recurrent/refractory malignant glioma	IL13-CD3ζ CD8+ cytotoxic T-lymphocyte clones	Intracranial	Transient inflammatory response, necrosis at tumor site, antigen loss	NCT00730613 [79]
2	IL3Rα2	I	Completed	Recurrent/refractory malignant glioma	IL-13 zetakine	Intratumoral infusion	No dose-limiting toxicity	NCT01082926 [80]
3	IL3Rα2	I	Ongoing	Recurrent/refractory malignant glioma	IL-13–4-1BBζ memory-derived T cells	Intracranial, intraventricular	Complete clinical response up to 7.5 months, antigen loss	NCT02208362 [82]
4	IL3Rα2	I	Ongoing	Leptomeningeal glioblastoma, ependymoma, or medulloblastoma	IL3Rα2-specific CAR with 4-1BB co-stimulation	Intraventricular	Evaluation of safety, feasibility, persistence, expansion	NCT04661384 [83]
5	IL3Rα2	I	Ongoing	Recurrent/refractory pediatric brain tumors	IL3Rα2-specific CAR	Intraventricular	Assessment of side effects, after lymphodepletion	NCT04510051 [84]
6	IL3Rα2	I	Ongoing	Glioblastoma	IL3Rα2-targeted CAR	Intraventricular, intratumoral	Trial for combination therapy with checkpoint inhibitor	NCT04003649 [134]
7	EGFRvIII	I, II	Completed	Malignant glioma	EGFRvIII-CD28–4-1BBζ Bulk T cells	Intravenous	Progression-free survival uup to12.5 months, dose-limiting toxicity at higher doses	NCT01454596 [86]
8	EGFRvIII	I	Terminated	Glioblastoma	EGFRVIII-4-1BBζ CAR	Intravenous	Increased IDO, FOXP3, IL-10, PD-L1 and TGFβ, antigen loss	NCT02209376 [90]
9	EGFRvIII	I	Completed	Glioblastoma	EGFrvIII-specific CAR T expressing 4-1BB and TCRζ	Intravenous	Result has not yet been published	NCT03726515 [92]
10	EGFRvIII	I	Terminated	Glioma grade IV	EGFRvIII CAR	Systemic	Radiolabelling of CAR T cells	NCT02664363 [136]
11	EGFRvIII	I	Terminated	Recurrent glioblastoma	EGFRvIII-targeted CAR	Intracerebral	Recruitment halted	NCT03283631 [174]
12	EGFR806	I	Ongoing	Recurrent/refractory EGFR+ pediatric CNS tumors	EGFR806-specific CAR	Delivered into tumor cavity or ventricular system	Evaluation of safety, efficacy, tolerability, distribution, tumor response	NCT03638167 [94]
13	EGFR806	I	Ongoing	Solid tumors including neuroblastoma	EGFR806-specific CAR	Systemic	Assessment of on target off tumor toxicity	NCT03618381 [95]
14	HER2	I	Completed	Glioblastoma	HER2-CD28ζ virus-specific T cells	Intravenous	No dose limiting toxicity	NCT01109095 [100]
15	HER2	I	Ongoing	Recurrent/refractory glioblastoma grade III/IV	HER2-specific, hinge-optimized, 4-1BB-co-stimulatory chimeric receptor	Intracerebral	Investigation on side effects and best suit dose	NCT03389230 [102]
16	HER2	I	Ongoing	Metastatic meningeal neoplasm	HER2-specific CAR	Intraventricular	Evaluation of side effects, best dose	NCT03696030 [103]
17	HER2	I	Ongoing	Recurrent/refractory HER2+ pediatric CNS tumors	HER2-specific CAR	Intracerebral	Evaluation of safety, efficacy, distribution	NCT03500991 [105]
18	HER2	I	Ongoing	HER2+ CNS tumors	HER2-specific CAR	Intracranial	Evaluation of efficacy, side effects, largest safe dose	NCT02442297 [106]
19	HER2	I	Ongoing	Pediatric recurrent/refractory ependymoma	HER2-specific CAR	Intravenous	Evaluation of safety and feasibility	NCT04903080 [107]
20	B7-H3	I, II	Ongoing	Recurrent/refractory glioblastoma	B7-H3-targeted CAR	Intratumoral, intraventricular	Concurrent therapy with temozolomide	NCT04077866 [111]
21	B7-H3	I	Ongoing	Diffuse intrinsic pontine glioma/diffuse midline glioma and recurrent or refractory pediatric central nervous system tumors	B7-H3-specific CAR	Delivered into tumor cavity or ventricular system	Assessment of safety, distribution, peripheral trafficking	NCT04185038 [112]
22	B7-H3	I	Unknown	Recurrent/refractory glioblastoma	B7-H3-targeted CAR	Intratumoral, intraventricular	Unknown status after May 2020	NCT04385173 [175]
23	CD147	I	Unknown	Malignant glioma	CD147 CAR T cells	Intracavity injection	Unknown status after May 2020	NCT04045847 [114]
24	GD2	I	Completed	Recurrent/refractory neuroblastoma	Anti-GD2 CAR	Systemic	No disease progression with detectable CAR levels upto 45 days	NCT02761915 [120]
25	GD2	I	Ongoing	GD2+ brain tumor	C7R-GD2.CAR T Cells	Intravenous	Ongoing safety and efficacy assessment	NCT04099797 [121]
26	GD2	I, II	Ongoing	Recurrent/refractory neuroblastoma	Anti-GD2 CAR T cells	Systemic	Dose escalation and expansion trial	NCT03373097 [122]
27	GD2	I	Ongoing	Intrinsic pontine glioma, spinal diffuse midline glioma	GD2-specific CAR	Intravenous	Assesment of safety, feasibility, recommendation of dose for phase II trial	NCT04196413 [123]
28	GD2	I	Ongoing	Pediatric neuroblastoma	GD2-specific CAR with autologous NKT cells expressing IL-15	Systemic	The first trial on GD2 expressing NKT cells	NCT03294954 [135]
29	EphA2	I, II	Withdrawn	Malignant glioma	EphA2-specific CAR	Systemic	Trial withdrawn	NCT02575261 [131]
30	CLTX	I	Ongoing	MMP2+ recurrent or progressive glioblastoma	CLTX (EQ)-CD28-CD3 zeta-CD19t-expressing CAR T lymphocytes	Intravenous	Tumor binding peptide used as targeting domain	NCT04214392 [127]
31	CD171	I	Ongoing	Neuroblastoma	CD171-specific CAR T cells expressing EGFR1	Systemic	Assessment for maximum tolerable dose	NCT02311621 [129]
32	NKG2D	I	Withdrawn	Solid tumors including glioblastoma	NKG2D-based CAR	Intravenous, intra-arterial	Trial withdrawn	NCT04270461 [142]
33	EGFRvIII, IL13Rα2, HER2, CD133, EphA2 or GD2	I	Ongoing	Recurrent malignant glioma	Biological, antigen-specific CAR T cells	Systemic delivery of lentiviral vector	Personalized CAR design based on tumor antigen expression	NCT03423992 [176]

Tumor tissue analyses demonstrated that antigen loss is a therapeutic escape mechanism [173]. Newer-generation CAR vectors came ahead with a prospect of overcoming antigen loss. The interactions between the host immune system and engineered T cells are also a matter of concern. According to clinical data, the tumor microenvironment responds to IL13Rα2 CAR T therapy by boosting CD3+, CD14+, and CD15+ immune cells as well as inflammatory cytokines after locoregional CAR infusion. Since CAR T therapy requires a highly expressed target throughout tumors and heterogeneity of tumor antigens leads to probable resistance towards CAR T therapy, the recognition of a broader pool of antigens is an urgent need.

The specificity of a few of the novel tumor-specific antigens under study raises significant questions. A potential concern regarding B7-H3 CAR T cells remains in the possibility of modulation of the inhibition of translation of B7-H3 mRNA by microRNAs during inflammation. This could potentially result in the expression of B7-H3 on healthy tissues, which would cause off-target toxicity [177]. While CD147 is overexpressed on malignant cells, it is still expressed at a low level on various normal tissues such as epithelial and endothelial cells of the brain and heart tissues [178]. Thus, there is a concern that CD147-specific CAR T cells may cause ‘on-target off-tumor’ side effects. Clinical outcomes are yet to reach a decisive verdict. Similarly, GD2 is expressed in normal brain tissues. Substantial CNS toxicity has been reported along with T cell infiltration into brain regions representing GD2-positive normal cells when GD2 CAR T cells were tested to treat neuroblastoma [179]. The future of CAR T cell engineering lies in abrogating their dependence on particular tumor antigen(s) and incorporating freedom to target a range of antigens, as well as giving them the ability to be turned on/off efficaciously. In order to address the challenges with CAR T cell-induced toxicity, inducible and/or programmable CAR systems and safety switches like suicide genes are appealing options when building novel strategies [180]. As discussed earlier, combination therapies may be a promising strategy to endorse CAR T cell activity and persistence. The clinical and molecular endpoints and correlating data that are gathered before, during, and after therapy can have an impact on future combination trials in order to comprehend the mechanisms of response and resistance to CAR-mediated treatment of brain cancer.

Several small molecule inhibitors have been discovered that can inhibit tumor growth, survival, angiogenesis, and metastasis by interfering with different transcription factors. Tyrosine kinase inhibitors have only had a modest therapeutic impact when used as monotherapy in clinical trials for glioblastoma [181]. However, tyrosine kinase inhibitors in combination with CAR T cells have demonstrated synergistic results in the treatment of other forms of solid tumors, making them a compelling candidate for glioblastoma testing [182, 183]. LB-100, a protein phosphatase 2A inhibitor, might augment the efficacy of CAIX-specific CAR T cells in the treatment of glioblastoma [184].

In many clinical studies, CAR T cells used as monotherapy have been found not to be sufficient to induce sustained clinical responses. However, one might still argue that the lack of clinical response to the treatment with CAR T cells alone is partially due to the recruitment of patients at advanced stages of the disease with a high tumor burden. An alternative therapeutic strategy with CAR T cells is the incorporation of a CAR construct into other types of effector immune cells such as NK cells. Oncolytic viruses can accelerate tumor lysis by selectively attacking cancer cells while sparing healthy cells, which can send out alarm signals and boost the immune systems [185]. In addition, oncolytic viruses may work in conjunction with CAR T therapy provided they are genetically transformed to express therapeutic transgenes endorsing a suppressive tumor microenvironment. Other promising combinatorial approaches include antagonistic antibodies specific to the 4-1BB co-stimulatory receptor, which can rapidly activate CAR T cells [186]. Vaccines containing glioma-associated antigens or dendritic cells loaded with mRNA or tumor lysate have been used to treat primary brain tumors, and they may work in tandem with CAR T therapy to overcome tumor heterogeneity and induce an endogenous immune response [187]. A combination of anti-podoplanin CAR T cells with oncolytic herpes virus G47D successfully reduced ‘on-target off tumor’ toxicity acting against patient-derived glioma stem cells while sparing normal cells [188].

Tumor irradiation acts somewhat as an in situ vaccine as it endorses the release of tumor-associated antigens that prompt antigen-presenting cells to migrate to draining lymph nodes, where they prime cytotoxic CD8+ T cells to generate an adaptive immune response including increased expression of T cell receptors [189]. Given that radiation plays a part in the recruitment of the adaptive immune response that is stimulated by the stimulator of the interferon gene (STING), it may also help with the trafficking and persistence issues of CAR T cells. Since the expansion of T cell receptors and activation of dendritic cells might result in ‘epitope spreading’ and immunologic memory against several tumor antigens, radiation and concurrent STING activation may also address post-CAR T antigen escape [190].

Patients with glioma have seen relatively modest success from CAR T cells, despite the fact that they have demonstrated exceptional effectiveness in the treatment of hematological malignancies [26]. Clearly, the ability of CAR T cell therapy to treat glioma is largely dependent on how the design of the cell constructs themselves are altered. CAR T cells can be significantly modified using contemporary genetic technologies like CRISPR/Cas9 in order to increase their therapeutic efficacy, persistence, and safety. Majority of glioma models employ immunosuppressed mice, which do not accurately replicate the tumor microenvironment to detect CAR T cell responses [191]. For the effective development of manipulation tactics, a deeper comprehension of the CNS environment in which CAR T cells function is important. Given how powerful immunosuppressive myeloid cells are and how they are present within the CNS, gene modifications intended to offset their effects may be supportive.

For the treatment of brain cancer, significant progress has been made with the identification of tumor-specific antigens and the development of cutting-edge CAR designs. Even though combinatorial targeting has shown promise in addressing tumor heterogeneity, research is still going on to find the ideal combination of targeted antigens. Combinatorial regimens that combine CAR therapeutics with immunomodulators and immune checkpoint inhibitors are likely to improve the effectiveness with which CAR T cell therapies can cause “epitope spreading” and so address the issue of antigen loss or antigen low escape. The majority of the confusion is caused by uncertainty regarding the dynamic changes tumors go through after an immune assault. Therefore, identifying the changes in intra-tumor subpopulations at the single-cell level following CAR therapy might direct CAR T cell therapy to properly address tumor heterogeneity. Overall, several strategies, including the insertion of cytokine transgenes, gene knock-out and knock-in, control of CAR production and activity, as well as multi-antigen targeting, show good promise in the field of anti-glioma CAR T cell treatment. The predicted advancements depend on continued development in removing the main obstacles to CAR T cell therapy of brain tumors i.e. tumor heterogeneity, T cell exhaustion, suppressive microenvironment, antigen escape, and insufficient T cell trafficking. An enhanced focus on the identification of ideal target antigens is an important area of future research that is required to achieve the potential of CAR T cell therapy for any form of solid tumors. The tumor surfaceome can now be completely cataloged to find a suitable set of differentially expressed cell surface antigens that can be incorporated into multi-specific CAR T cells. Efforts to boost tumor sensitivity to CAR therapy by antigen modulation may also improve outcomes in the long run, but they must be carefully assessed for potential off-target effects. Exhaustion-resistant CAR T cells need to be designed to produce a powerful, long-lasting cytotoxic response. Developing ways to make T cells exhaustion-resistant will significantly improve the efficacy of adoptive cell treatments. Measures to minimize exhaustion are also likely to limit the impact of inhibitory signals produced by the tumor microenvironment.

## Conclusion and outlooks

CAR T cells could be a promising therapeutic option for brain cancer since so many potential target tumor antigens have been identified so far. However, one of the biggest hurdles in this field is choosing a suitable antigen to target, especially one that is expressed in brain cancer and brain cancer-initiating cells and destroys them without harming normal brain cells. Understanding the optimal trafficking of CAR T cells to brain tumors is still to be clearly interpreted. Though most clinical trials have demonstrated that CAR T cells as a monotherapy are often ineffective in cases of solid tumors due to their immune escape mechanisms, the efficacy and scope of CAR T cell therapies are continually being expanded, and innovative approaches are being explored to improve safety as well as efficacy. These innovative approaches would lead to the wider applications of this technology for the efficient management of brain cancer through the optimization of CAR T cell biology and others. However, concerns with CAR T cells also spin around their cost and scale-up, which also require substantial attention.

## Abbreviations

CAR T cells: Chimeric antigen receptor T cells
CNS: Central nervous system
CARs: Chimeric antigen receptors
MHC: Major histocompatibility complex
BBB: Blood-brain barrier
BBTB: Blood-brain tumor barrier
TCR: T cell receptor
TRAC: T cell receptor α constant
FDA: Food and drug administration
